# Targeting SWI/SNF ATPases in H3.3K27M diffuse intrinsic pontine gliomas

**DOI:** 10.1073/pnas.2221175120

**Published:** 2023-04-24

**Authors:** Mateus Mota, Stefan R. Sweha, Matt Pun, Siva Kumar Natarajan, Yujie Ding, Chan Chung, Debra Hawes, Fusheng Yang, Alexander R. Judkins, Susanta Samajdar, Xuhong Cao, Lanbo Xiao, Abhijit Parolia, Arul M. Chinnaiyan, Sriram Venneti

**Affiliations:** ^a^Laboratory of Brain Tumor Metabolism and Epigenetics, Department of Pathology, University of Michigan, Ann Arbor, MI 48109; ^b^Chad Carr Pediatric Tumor Center, Department of Pediatrics, University of Michigan, Ann Arbor, MI 48109; ^c^Cellular and Molecular Biology Program, University of Michigan Medical School, Ann Arbor, MI 48109; ^d^Medical Scientist Training Program, University of Michigan Medical School, Ann Arbor, MI 48109; ^e^Department of New Biology, Daegu Gyeongbuk Institute of Science and Technology, Daegu 42988, Korea; ^f^Department of Pathology and Laboratory Medicine, Children’s Hospital Los Angeles, Keck School of Medicine University of Southern California, Los Angeles, CA 90027; ^g^Aurigene Discovery Technologies, Bengaluru, Karnataka 560100, India; ^h^Michigan Center for Translational Pathology, Department of Pathology, University of Michigan Medical School, Ann Arbor, MI 48109; ^i^Rogel Cancer Center, University of Michigan Medical School, Ann Arbor, MI 48109; ^j^Department of Urology, University of Michigan Medical School, Ann Arbor, MI 48109; ^k^HHMI, University of Michigan Medical School, Ann Arbor, MI 48109

**Keywords:** pediatric brain cancer, H3K27M mutation, SWI/SNF complex

## Abstract

Diffuse midline gliomas (DMGs) including diffuse intrinsic pontine gliomas (DIPGs) bearing lysine-to-methionine mutations in histone H3 at lysine 27 (H3K27M) are lethal childhood brain cancers. These tumors harbor a global reduction in the transcriptional repressive mark H3K27me3 accompanied by an increase in the transcriptional activation mark H3K27ac. We postulated that H3K27M mutations, in addition to altering H3K27 modifications, reprogram the master chromatin remodeling switch/sucrose nonfermentable (SWI/SNF) complex. The SWI/SNF complex can exist in two main forms termed BAF and PBAF that play central roles in neurodevelopment and cancer. Moreover, BAF antagonizes PRC2, the main enzyme catalyzing H3K27me3. We demonstrate that H3K27M gliomas show increased protein levels of the SWI/SNF complex ATPase subunits SMARCA4 and SMARCA2, and the PBAF component PBRM1. Additionally, knockdown of mutant H3K27M lowered SMARCA4 protein levels. The proteolysis targeting chimera (PROTAC) AU-15330 that simultaneously targets SMARCA4, SMARCA2, and PBRM1 for degradation exhibits cytotoxicity in H3.3K27M but not H3 wild-type cells. AU-15330 lowered chromatin accessibility measured by ATAC-Seq at nonpromoter regions and reduced global H3K27ac levels. Integrated analysis of gene expression, proteomics, and chromatin accessibility in AU-15330-treated cells demonstrated reduction in the levels of FOXO1, a key member of the forkhead family of transcription factors. Moreover, genetic or pharmacologic targeting of FOXO1 resulted in cell death in H3K27M cells. Overall, our results suggest that H3K27M up-regulates SMARCA4 levels and combined targeting of SWI/SNF ATPases in H3.3K27M can serve as a potent therapeutic strategy for these deadly childhood brain tumors.

## Significance

H3K27M mutant DMGs including DIPGs are deadly childhood brain cancers. More than 250 clinical trials over the past 50 y have failed to yield effective therapies. This underscores the importance of understanding the biology of these tumors to develop efficacious therapies. Because H3K27M mutations suppress PRC2 function, and PRC2 opposes the BAF form of the SWI/SNF complex, we targeted key members of the SWI/SNF complex as a potential therapy. We demonstrate that a PROTAC tool compound AU-15330 that simultaneously degrades the ATPases SMARCA4 and SMARCA2 and PBRM1 selectively kills H3.3K27M but not H3WT glioma cell lines. Our studies suggest that targeting key components of the SWI/SNF complex can provide a potent therapy for these lethal pediatric brain cancers.

H3K27M diffuse midline gliomas (DMGs), including diffuse intrinsic pontine gliomas (DIPGs), are lethal childhood brain tumors. The available treatment options, chemo- and radiotherapy, are ineffective, and over 90% of patients die within 1.5 y of diagnosis (1, 2). These tumors harbor missense mutations in histone 3-encoding genes that result in a lysine-to-methionine substitution at position 27 mainly in genes *H3-3A* (termed H3.3K27M) and to a lesser extent in *H3C2* (termed H3.1K27M, collectively referred to as H3K27M) (3–5). H3K27M mutations result in global reduction of the repressive H3K27me3 mark, accompanied by aberrant H3K27ac-enriched enhancers and superenhancers and deregulation of gene expression. Additionally, abnormal genomic distribution of other histone marks, including H3K4me3, H3K36me3, H3K36me2, and H2K119ub, has been reported (6–15). These observations have led to the proposal of inhibitors of factors that mediate histone posttranslational modifications for potential therapies, including inhibitors of H3K27-demethylases, HDACs, EZH2, BET proteins, LSD1, and BMI1 (14–21).

However, it is not known whether H3K27M mutations impact chromatin by altering other epigenetic regulators in addition to histone modifications and whether this information can be leveraged for designing therapeutics. The SWI/SNF complex is a master epigenetic modulator that facilitates nucleosome incorporation and displacement by sliding or evicting histone octamers, resulting in differential chromatin accessibility to transcription factors (22, 23). The complex can exist in two forms called BAF and PBAF with several common, unique, and obligate members. BAF is critical to establish enhancers and superenhancers (24–26). On the contrary, PBAF localizes to active promoters and is defined by the expression of three components—PBRM1, ARID2, and BRD7. The SWI/SNF complexes are ATP dependent with two main ATPase subunits—SMARCA4 (BRG1) and SMARCA2 (BRM). Several of these key subunits, including the ATPases, are mutated in various tumors, implicating a central role of the SWI/SNF complex in cancers in general (22, 23).

Global H3K27me3 reduction is mediated by H3K27M mutations that suppress the functions of the polycomb repressive complex 2 (PRC2) which contains the H3K27-specific methyltransferase EZH2 (6–8, 27, 28). PRC2 function can also be antagonized by BAF. This was first established in rhabdoid tumors bearing SMARCB1 loss-of-function mutations (29). Subsequent studies have shown that recruitment of BAF complexes to enhancers evicts PRC2 to lower H3K27me3 levels and enables genomic enrichment of H3K27ac (24–26, 29–32). Moreover, suppression of EZH2 is proposed as a therapeutic strategy for both SMARCB1 mutant rhabdoid tumors and H3K27M gliomas (20, 21, 29–31). Because H3K27M similarly antagonizes the function of PRC2, we hypothesized that the SWI/SNF complex plays an important role in H3K27M DMGs. We employed a proteolysis-targeting chimera (PROTAC) degrader compound, AU-15330, that specifically degrades SMARCA4, SMARCA2, and PBRM1. Enhancer-addicted prostate cancers are potently suppressed by AU-15330 (33). Since H3K27M tumors are also enhancer addicted (9, 19, 34, 35), we hypothesized that AU-15330 would show selective efficacy in H3K27M as compared to H3WT tumor cells. We addressed this hypothesis using an integrated approach encompassing proteomics and next-generation sequencing.

## Results

### SMARCA4 Protein Levels Are Higher in H3.3K27M Compared to H3WT Cells.

**Fig. 1.**
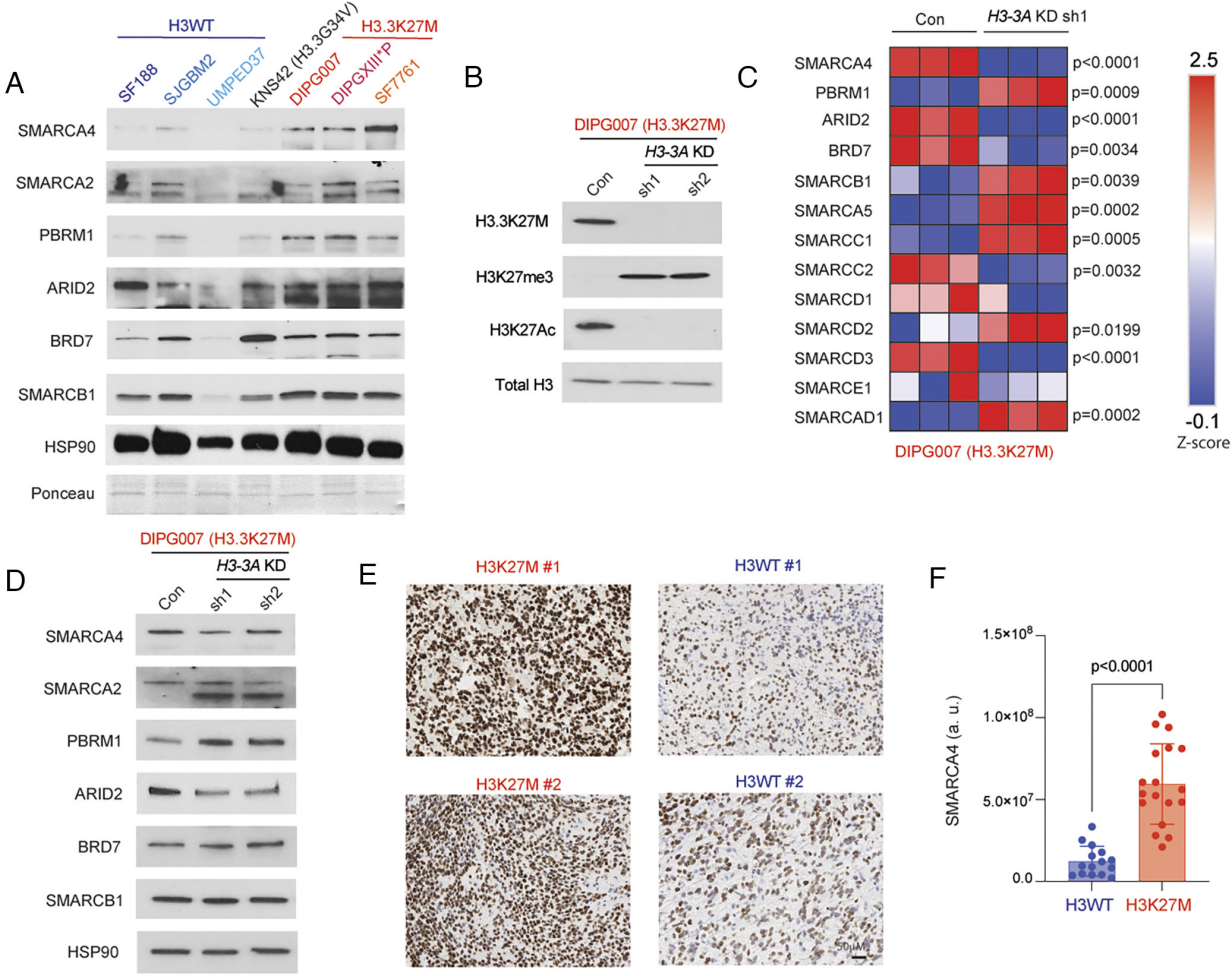
SMARCA4 protein levels are higher in H3.3K27M compared to H3WT cells. (*A*) Immunoblots of patient-derived H3 WT (SF188, SJGBM2, and UMPED37), H3.3G34V (KNS42), or H3.3K27M (DIPG007, DIPGXIII*p, and SF7761) cell lines probed for SMARCA4, SMARCA2, PBRM1, ARID2, BRD7, and SMARCB1; western blot for HSP90 and Ponceau staining were used as loading control. (*B*) Immunoblots of DIPG007 cells with or without stable knockdown (KD) of *H3-3A* with two independent shRNAs (sh1 or sh2) probed for H3.3K27M, H3K27me3, and H3K27ac; total H3 was probed as loading control. (*C*) Heatmap of normalized protein abundance (Z-score) of key components of BAF and PBAF (PBRM1, ARID2, and BRD7) SWI/SNF complex in DIPG007 H3.3K27M with or without *H3-3A* KD acquired by untargeted proteomics (n = 3, each). (*D*) Immunoblots of DIPG007 cells with or without *H3-3A* KDs from B probed for SMARCA4, SMARCA2, PBRM1, ARID2, BRD7, and SMARCB1. HSP90 was probed as loading control. (*E*) Representative images of H3K27M and H3WT (2 cases each) tumors stained for SMARCA4 by immunohistochemistry. (Scale bar, 50 μM.) (*F*) Matlab-based quantification of SMARCA4 (*Y* axis, a.u.=arbitrary units determined by number of pixels × pixel intensity; three randomly selected regions/case) in H3WT (n = 5) and H3K27M (n = 6) DMGs. Data were analyzed by two-sided, two-tailed, nonpaired *t* test.

We began by assessing the levels of various SWI/SNF-associated proteins including the ATPases SMARCA4 and SMARCA2; PBAF-associated members PBRM1, BRD7, and ARID2; and SMARCB1 in a panel of H3WT (SF188, SJGBM2, and UMPED37), H3.3G34V (KNS42), and H3.3K27M (DIPG007, DIPGXIII*P, SF7761, and BT425) low-passage, patient-derived cell lines. Overall, we noted higher expression of SMARCA4, SMARCA2, and PBRM1 in H3.3K27M versus H3WT and H3.3G34V cell lines (Fig. 1*A*). We next knocked down (KD) *H3-3A* to reduce mutant H3.3K27M levels. This was accompanied by an increase in H3K27me3 and lowered H3K27ac levels (Fig. 1*B*). We determined changes in various SWI/SNF complex members by proteomics on H3.3K27M KD in DIPG007 cells. SMARCA4, ARID2, BRD7, SMARCC2, and SMARCD3 protein levels were significantly reduced (Fig. 1*C*). On the contrary, PBRM1, SMARCB1, SMARCA5, SMARCC1, SMARCD3, and SMARCD1 levels were significantly increased (Fig. 1*C*). We validated key proteomic findings by western blotting in DIPG007 cells with both shRNAs. We did not observe similar consistent changes in ARID2, BRD7, and SMARCB1 (Fig. 1*D*); however, reduction in SMARCA4 and increased PBRM1 were confirmed (Fig. 1*D*). We next performed immunohistochemistry (IHC) for SMARCA4 in DMG tumor samples (Fig. 1 *E* and *F*). IHC confirmed increased SMARCA4 levels in H3K27M compared to H3WT DMGs as observed in patient-derived cell lines (Fig. 1 *E* and *F*). Expression (mRNA) levels of Smarca4, Smarca2, and Pbrm1 were not altered in isogenic mouse neuronal stem cells bearing H3.3K27M versus H3.3WT from our previous studies (*SI Appendix*, Fig. S1*A*) (36). This suggests that H3K27M may not alter expression but could impact stabilization of the SWI/SNF complex as suggested by others (37, 38).

### H3.3K27M Cell Lines Are Sensitive to SMARCA2, SMARCA4, and PBRM1 Protein Degradation Induced by Treatment with the PROTAC Degrader AU-15330.

**Fig. 2.**
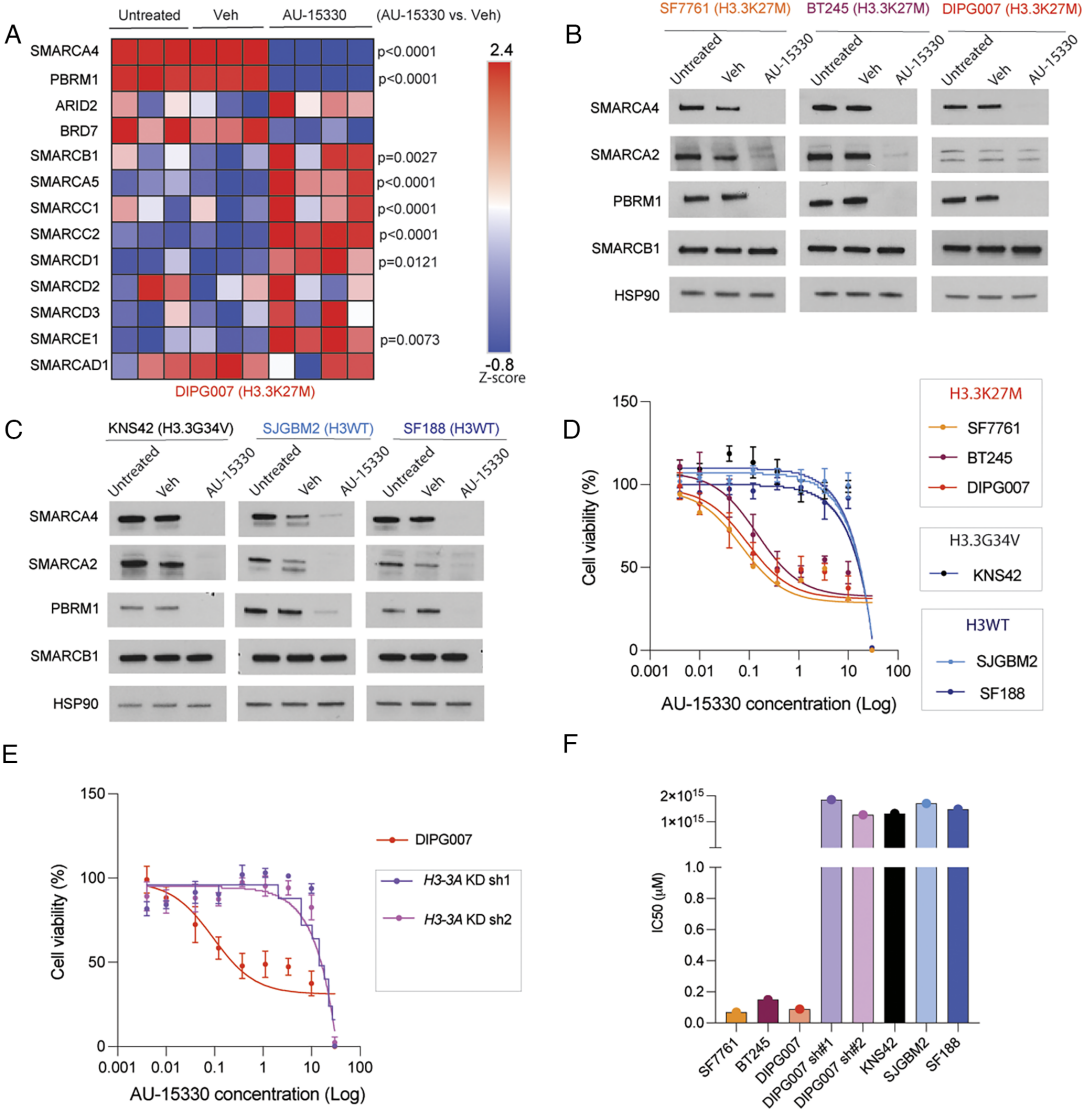
H3.3K27M cell lines are sensitive to SMARCA2, SMARCA4, and PBRM1 protein degradation using the PROTAC AU-15330. (*A*) Heatmap of normalized protein abundance (Z-score) of key SWI/SNF complex components in untreated DIPG007 cells (n = 3) or DIPG007 cells treated with AU-15330 PROTAC (n = 4, 1 µM for 24 h) or vehicle (Veh, DMSO, n = 3), acquired by untargeted proteomics. Data were analyzed by ANOVA; *P* values indicated are between Veh and AU-15330-treated cells. (*B*) Immunoblots of H3.3K27M mutant cell lines (SF7761, BT245, and DIPG007) treated with AU-15330 (1 µM for 24 h) or Veh and probed for SMARCA4, SMARCA2, PBRM1, and SMARCB1. HSP90 was probed as loading control. (*C*) Immunoblots of H3.3G34V mutant (KNS42) and H3WT (SJGBM2 and SF188) cell lines treated with AU-15330 (1 µM for 24 h) or Veh and probed for SMARCA4, SMARCA2, PBRM1, and SMARCB1. HSP90 was probed as loading control. (*D*) Cell viability (normalized to Veh, percentage, *Y* axis) of H3WT (SJGBM2 and SF188), H3.3G34V mutant (KNS42), and H3.3K27M mutant (SF7761, BT245, and DIPG007) cell lines on treatment with different concentrations of AU-15330 (log concentrations, *X* axis) for 5 d (n = 3 for each concentration/cell line). (*E*) Cell viability (normalized to Veh, percentage, *Y* axis) of DIPG007 cells with or without for *H3-3A* KD with two independent shRNAs (*H3-3A* sh1 or sh2) treated with different concentrations of AU-15330 (log concentrations, *X* axis) for 5 d (n = 3 for each concentration/cell line). (*F*) Half maximal inhibitory concentration (IC_50_) of AU-15330 (μM=Molar, *Y *axis) for H3.3K27M mutant (SF7761, BT245, and DIPG007), *H3-3A* KD sh1 and sh2 DIPG007, H3.3G34V mutant (KNS42), and H3WT (SJGBM2 and SF188) cells based on cell viability curves in (*D*) and (*E*).

Since SMARCA4, SMARCA2, and PBRM1 levels were elevated in H3.3K27M versus H3WT and H3.3G34V cell lines, we sought to determine the effects of a PROTAC tool compound (AU-15330) designed to simultaneously target all the three components. DIPG007 cells treated with AU-15330 showed reduced levels of SMARCA4 and PBRM1 by proteomics (Fig. 2*A*) and immunoblotting (Fig. 2*B* and *SI Appendix*, Fig. S1*B*). Additionally, proteomics showed a significant increase in SMARCB1, SMARCA5, SMARCC1, SMARCC2, SMARCD1, and SMARCE1 (Fig. 2*A*). We tested the effects of AU-15330 in our panel of H3.3K27M, H3WT, and H3G34V cells lines. All cell lines showed lowered SMARCA4, SMARCA2, and PBRM1 levels (Figs. 2 *B* and *C* and *SI Appendix*, Fig. S1*B*). However, H3.3K27M cells showed far greater sensitivity to AU-15330 as compared to H3WT and H3G34V cell lines (Fig. 2*D* and *SI Appendix*, Fig. S1 *C* and *D*). Moreover, knockdown of mutant H3.3K27M using our two independent shRNAs rendered H3.3K27M DIPG007 cells insensitive to AU-15330 (Fig. 2*E* and *SI Appendix*, Fig. S1*E*). Importantly, AU-15330 IC_50_ values were 15-fold lower in H3.3K27M as compared to H3WT, or H3.3G34V (KNS42), and DIPG007 with H3.3K27M KD with both shRNAs (Fig. 2*F*). These data demonstrate that H3.3K27M cells are far more sensitive to AU-15330 as compared to H3WT and H3.3G34V mutant cell lines.

### AU-15330 Reduces Chromatin Accessibility at Nonpromoter Regions in H3.3K27M Mutant Cells.

**Fig. 3.**
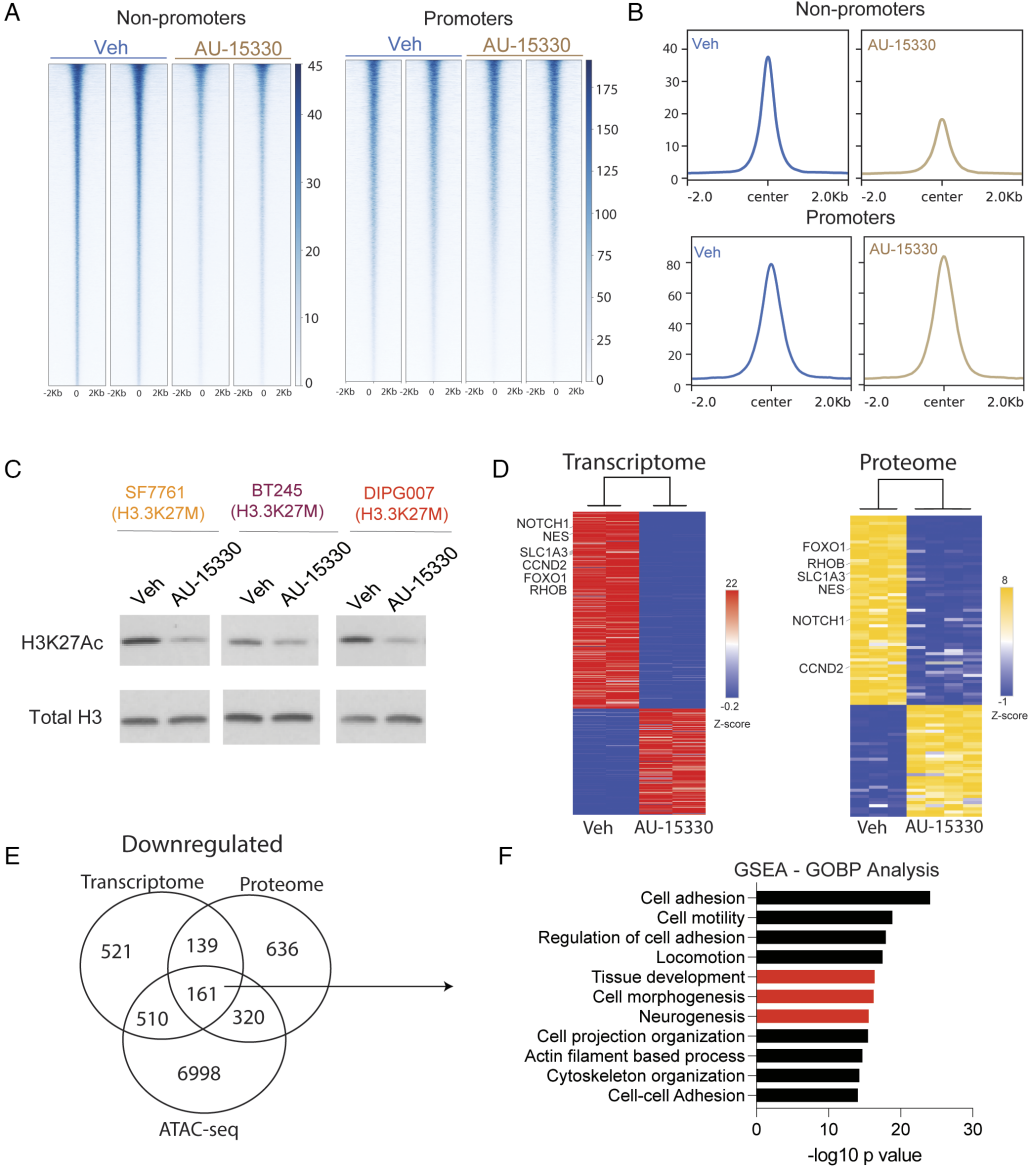
AU-15330 reduces chromatin accessibility at nonpromoter regions in H3.3K27M mutant cells. (*A*) Heat maps of peak intensity (plotted as ± 2.0 kb from peak center) and overall peak representation of nonpromoter regions and promoters (plotted as ± 2.0 kb from center, transcriptional start site). (*B*) Overall peak representation of nonpromoter and promoter regions from 3A. (*C*) Immunoblots of H3.3K27M mutant cell lines (SF7761, DIPG007, and BT245) treated with AU-15330 (1 µM for 24 h) or Veh and probed for H3K27Ac. Total H3 was probed as loading control. (*D*) Heatmap of differentially expressed genes (*Left*, RNA-Seq) and proteins (*Right*, untargeted proteomics) in DIPG007 cells treated with AU-15330 (1 µM for 24 h) or Veh. Red = up-regulated genes, yellow = up-regulated proteins, and blue = down-regulated genes or proteins (RNA-Seq n = 2/condition; proteomics: n = 3 for Veh and n = 4 for AU-15330). (*E*) Venn diagram depicting overlap of genes with lowered chromatin accessibility (ATAC-Seq), lowered expression by RNA-Seq, and proteomics in DIPG007 cells treated with AU-15330 versus Veh. (*F*) Pathway analysis of 161 genes from Venn diagram. Red bars indicate pathways related to development.

We next elucidated genome-wide changes in chromatin accessibility with AU-15330 treatment. DIPG007 cells were treated with AU-15330 (1 µM) or vehicle for 24 h and profiled for genome-wide changes by ATAC-Seq. We noted marked lowering of chromatin accessibility at gene bodies, introns, and distal intergenic regions (*SI Appendix*, Fig. S2 *A–C*). These overlapped mainly to nonpromoter genomic areas, while promoter regions were largely unaltered (Fig. 3 *A* and *B* and Dataset S1). This corresponded to a reduction in global H3K27ac levels in all the three H3.3K27M cell lines (Fig. 3*C*). In contrast, overall levels of H3K4me1, H3K4me3, and H3K27me3 remained unaltered even with high concentrations of AU-15330 (10 µM for 48 h, *SI Appendix*, Fig. S2*D*). Moreover, H3.3K27M levels were unchanged with AU-15330 treatment (*SI Appendix*, Fig. S2*D*). To determine the functional significance of changes in chromatin accessibility, we performed RNA-Seq (Dataset S2) and proteomics (Dataset S3) in parallel in H3.3K27M cells with or without treatment with AU-15330 (1 µM for 24 h) (Fig. 3*D*). We overlapped down-regulated genes from RNA-Seq and proteomics that also showed lowered chromatin accessibility to identify 161 commonly down-regulated genes (Fig. 3*E* and Dataset S4). Gene set enrichment analysis (GSEA) of these 161 genes showed downregulation of pathways related to cell adhesion, cell motility, cell morphogenesis, and neurogenesis (Fig. 3*F*).

### Chromatin Accessibility, Gene Expression, and Protein Abundance of FOXO1 Are Decreased by AU-15330.

**Fig. 4.**
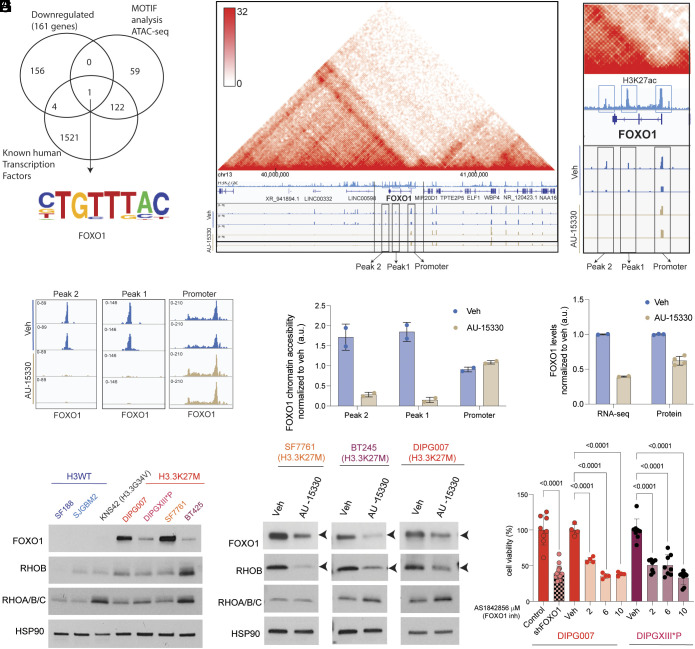
Chromatin accessibility, gene expression, and protein abundance of FOXO1 are lowered by AU-15330 PROTAC. (*A*) Venn diagram depicting overlap of 161 down-regulated genes from Fig. 3*F*, with motif analysis of genes with lowered chromatin accessibility by ATAC-Seq, and all known human transcription factor genes. FOXO1 motif is indicated. (*B*) Hi-C heatmap (*Left*) depicting a topologically associating domain (TAD) between the *FOXO1* promoter and ATAC-Seq peaks 1 and 2 associated with the *FOXO1* locus in DIPG007 cells treated with AU-15330 (1 µM for 24 h, tan, n = 2) or Veh (blue, n = 2). H3K27ac (pale blue) track from DIPG007 shows localizations of peaks 1 and 2 with H3K27ac-enriched FOXO1 enhancer sites (pale blue boxes). Magnified FOXO1 locus (rectangle, right) is illustrated. (*C* and *D*) Representation (*C*) and quantification (*Y* axis, a.u. = arbitrary units normalized to Veh) (*D*) of ATAC-Seq peak tracks/chromatin accessibility at the *FOXO1* promoter and at peak 1 and 2 associated with the *FOXO1* locus from (*B*). (*E*) FOXO1 mRNA and protein levels (a.u. = arbitrary units normalized to Veh) of DIPG007 cells treated with or without AU-15330 by RNA-Seq (n = 2/condition) and proteomics (n = 3 for Veh; n = 4 for AU-15330). (*F*) Immunoblots of H3WT (SF188 and SJGBM2), H3.3G34V mutant (KNS42), and H3.3K27M mutant cell lines (SF7761, DIPGXIII*P, BT245, and DIPG007) probed for FOXO1, RHOB, and RHOA/B/C. HSP90 was probed as loading control. (*G*) Immunoblots of H3.3K27M mutant cell lines (SF7761, BT245, and DIPG007) treated with AU-15330 (1 µM for 24 h) or Veh probed for FOXO1, RHOB, and RHOA/B/C. HSP90 was probed as loading control. Arrows indicate FOXO1 and RHOB. (*H*) Cell viability assessment (normalized to Veh, percentage, *Y *axis) of H3.3K27M mutant cell lines DIPG007 with (n=16) or without (n = 8) *FOXO1* knock down and DIPG007 (n = 4) and DIPGXIII*p (n = 8) cells treated with the FOXO1 inhibitor AS-1842856 (2, 6, or 10 µM for 48 h) or Veh.

We performed an integrated analysis on AU-15330 treatment using motif analyses on genes with lowered chromatin accessibility along with the 161 functionally down-regulated genes (Fig. 3*E*) and cross referenced these data with known human transcription factors. These analyses identified *FOXO1* (Forkhead Box O1), a key forkhead master transcriptional factor down-regulated by AU-15330 (Fig. 4*A* and Dataset S5). Reduced chromatin accessibility was noted at two peaks corresponding to H3K27ac-marked enhancers at the *FOXO1* locus in DIPG007 cells (Fig. 4 *B–D*). In contrast, chromatin accessibility at the *FOXO1* promoter was not altered with AU-15330 treatment (Fig. 4 *B–D*). Importantly, Hi-C data showed a topologically associated domain formed between the *FOXO1* promoter region, the two downstream enhancer peaks, and downstream intergenic regions comprised of *LINC00598* and *LINC00332* (*SI Appendix*, Fig. S3*A* and Fig. 4*B*). Lowered chromatin accessibility at the *FOXO1* locus was accompanied by reduction of both *FOXO1* mRNA and protein levels (Figs.3*D* and 4*E*) and downregulation of canonical *FOXO1* targets by RNA-Seq (*SI Appendix*, Fig. S3*B*). Of the FOXO1 targets, we identified downregulation of Ras homolog (RHO) family GTPases (*SI Appendix*, Fig. S3 *C* and *D*). Among the RHO family GTPases, RHOB was one of the 161 genes that were down-regulated by RNA-Seq, proteomics, and ATAC-Seq in AU-15330 versus vehicle-treated DIPG007 cells (Fig. 3 *D–F*). Therefore, we focused our efforts on assessing the expression of FOXO1 and RHOB in our panel of cells. Both FOXO1 and RHOB protein levels were expressed at higher levels in H3.3K27M versus H3WT or H3G34V cells (Fig. 4*F*). Importantly, both FOXO1 and RHOB protein levels were reduced upon AU-15330 treatment in all the three H3.3K27M cell lines (Fig. 4*G*). In contrast, AU-15330 versus vehicle treatment did not show consistent FOXO1 or RHOB alterations in H3WT and H3G34V cells (*SI Appendix*, Fig. S3*E*). Finally, both genetic and pharmacologic (FOXO1 inhibitor AS1842856) suppression of FOXO1 resulted in cell death in H3.3K27M cells (Fig. 4*H* and *SI Appendix*, Fig. S3*F*). AS1842856 binds to active FOXO1, but not the Ser256-phosphorylated form (39–41). Accordingly, AS1842856 did not show a consistent pattern on p-ser256-FOXO1 levels in H3.3K27M cells (*SI Appendix*, Fig. S3*G*). Overall, these results suggest that AU-15330 toxicity is, in part, mediated by downregulation of FOXO1.

## Discussion

The discovery of H3K27M mutations in DIPGs and DMGs has brought chromatin biology to the forefront to define epigenetic treatments for these lethal tumors. Due to inhibition of the function of the PRC2 complex, H3K27M mutations result in global reduction of H3K27me3. This is accompanied by abnormal genomic distribution of other histone posttranslational marks, including H3K27ac, H3K4me3 (including bivalent H3K4me3/H3K27me3), H3K36me2, H3K36me3, and H2AK119ub. To further define aberrant chromatin in H3K27M tumors, we interrogated key members of the SWI/SNF complex, including the ATPases SMARCA4 and SMARCA2, and key components of PBAF, including PBRM1, BRD7, and ARID2. All the tested components were higher in H3.3K27M versus H3WT and H3.3G4V cell lines. Upon knockdown of H3.3K27M, we noted a reduction in SMARCA4 accompanied by an increase in PBRM1 levels. In support of these data, previous studies using DNA-barcoded H3.3K27M containing nucleosomes suggest mutant histone interactions with both BAF and PBAF (37).

In this study, we utilized a PROTAC compound, AU-15330, that simultaneously degrades SMARCA4, SMARCA2, and PBRM1 and is toxic in enhancer-addicted prostate cancers (33). H3K27M tumors show elevated H3K27ac levels and are also enhancer addicted (9, 19, 34, 35). Accordingly, we noted selective toxicity in H3.3K27M versus H3WT and H3.3G34V cell lines accompanied by a reduction in global H3K27ac levels. AU-15330 lowered chromatin accessibility of genes at nonpromoter regions, and functional RNA-Seq and proteomic analyses showed downregulation of developmentally related pathways, cell adhesion, and cell locomotion. Similar results upon targeting SMARCA4 in H3K27M tumors were also observed by two independent groups while our manuscript was in preparation (42, 43). While our experiments were focused on H3.3K27M tumor cells, both these studies show similar effects on H3.1K27M cells, suggesting that targeting the SWI/SNF complex may be a key therapeutic strategy for H3.3/H3.1K27M DIPGs and DMGs.

Our approach of integrating motif analysis with down-regulated transcription factors on treatment with AU-15330 identified the master transcriptional regulator FOXO1. Lowered chromatin accessibility was noted at regions corresponding to *FOXO1* enhancers in a topologically associating domain formed at the *FOXO1* promoter. FOXO1 is important for brain development and deregulated in several cancers, including gliomas (37, 44–48). Alveolar rhabdomyosarcomas are driven by PAX3–FOXO1 and PAX7–FOXO1 fusion proteins in ~80% of cases (49, 50). Intriguingly, PAX3/7–FOXO1 fusion proteins epigenetically reprogram SMARCA4, resulting in aberrant enhancer activation that creates a therapeutic dependency with SMARCA4 inhibitors (51, 52). FOXO1 levels were decreased by AU-15330, and corresponding downregulation of FOXO1 canonical pathways was noted, including lowering of RHOB levels in H3K27M but not H3WT cells. Rho GTPases, including RHOB, can regulate several pathways related to signal transduction, cell adhesion, tumor cell invasion, and cell division (53), warranting further future examination of both FOXO1 and RHOB in H3K27M DMGs. However, AU-15330 down-regulated multiple pathways related to cell adhesion, cytoskeletal organization, and morphogenesis, suggesting that toxicity arises from multiple mechanisms that are both FOXO1 dependent and independent.

In summary, we note that H3.3K27M gliomas show alterations in key components of both the BAF and PBAF SWI/SNF complex. Therapeutically, a PROTAC compound simultaneously targeting SMARCA4, SMARCA2, and PBRM1 showed selective toxicity in H3.3K27M in our panel of tested cell lines. Our results suggest that targeting central epigenetic regulators including SMARCA4 may be a key therapeutic target for H3K27M DIPGs and DMGs, warranting further biologic and preclinical in vivo studies.

## Materials and Methods

### Patient-Derived Cell Lines.

Cell lines were cultured in a 5% CO2-humidified incubator at 37 °C. BT245 (obtained from Nada Jabado, University of Montreal), DIPG-007 (HSJD-DIPG007, obtained from Rintaro Hashizume, Northwestern University; RRID: CVCL_VU70), and DIPG-XIII*p (obtained from Michelle Monje, Stanford University; RRID: CVCL_IT41) H3.3K27M cells were cultured in Neurobasal-A (Thermo Fisher Scientific #10888022) and DMEM/F-12 (Thermo Fisher Scientific #11330032) media supplemented with HEPES (1 M) (Thermo Fisher Scientific #15630080), sodium pyruvate (100 mM) (Thermo Fisher Scientific #11360070), MEM nonessential amino acid solution (100X) (Thermo Fisher Scientific #11140050), GlutaMAX™ supplement (Thermo Fisher Scientific #35050061), B-27™ supplement (50X), minus vitamin A (Thermo Fisher Scientific #12587010), 20 ng/μL human EGF (Shenandoah #100-26, Warminster, PA), 20 ng/μL human FGF-BASIC-154aa (Shenandoah #100-146), 10 ng/μL human PDGF-AA (Shenandoah #100-16), 10 ng/μL human PDGF-BB (Shenandoah #100-18), 2 µg/mL 0.2% heparin solution (StemCell Technologies, Inc. #07980, Cambridge, MA), and antibiotic–antimycotic (100X) (Thermo Fisher Scientific #15240096). SF7761 H3.3K27M cells (obtained from Rintaro Hashizume, Northwestern University; RRID: CVCL_IT45) were cultured in Neurobasal-A (Thermo Fisher Scientific #10888022) media supplemented with serum free N-2 supplement (100X) (Thermo Fisher Scientific #17502048), B-27™ supplement (50X) (Thermo Fisher Scientific #17504044), L-glutamine (200 mM) (Thermo Fisher Scientific #A2916801), 7.5% albumin, bovine fraction V (BSA) (dot scientific, inc #DSA30075, Burton, MI), 20 ng/μL human EGF (Shenandoah #100-26), 20 ng/μL human FGF-BASIC-154aa (Shenandoah #100-146), 2 µg/mL heparin solution, 0.2% (StemCell Technologies, Inc. #07980), and penicillin–streptomycin (10,000 U/mL) (Thermo Fisher Scientific #115140122). UMPed37 H3 WT (obtained from Carl Koschmann, University of Michigan) and KNS42 H3.3G34V (obtained from Carl Koschmann, University of Michigan; RRID: CVCL_0378) cells were cultured in DMEM/F-12 (Thermo Fisher Scientific #11320033) supplemented with GlutaMAX™ supplement (Thermo Fisher Scientific #35050-061), 10% fetal bovine serum (VWR #89510-186, Radnor PA), Normocin™ (InvivoGen #ant-nr-1), and antibiotic–antimycotic (100X) (Thermo Fisher Scientific #15240-096). SF188 (obtained from Craig B. Thompson, Memorial Sloan Kettering Cancer Center; RRID: CVCL_6948) and HEK293T (obtained from Andrew Lieberman, University of Michigan) H3 WT cells were cultured in DMEM, high glucose, no glutamine (Thermo Fisher Scientific #111960-044) supplemented with 10% fetal bovine serum (VWR #89510-186), L-glutamine (200 mM) (Thermo Fisher Scientific #A2916801), and penicillin–streptomycin (10,000 U/mL) (Thermo Fisher Scientific #115140122). SJGBM2 H3 WT cells (obtained from Carl Koschmann, University of Michigan; RRID: CVCL_M141) were cultured in IMDM (Thermo Fisher Scientific #112440-053) supplemented with 20% fetal bovine serum (VWR #89510-186), Normocin™ (InvivoGen #ant-nr-1), and antibiotic–antimycotic (100X) (Thermo Fisher Scientific #15240-096).

### Chemical Compounds.

AU-15330 proteolysis-targeting chimera (PROTAC) compound was provided by Aurigene Biosciences. Cells were treated with 1 µM AU-15330 for 24 h, unless otherwise noted. AS-1842856 (#16761) was purchased from Cayman Chemical, and cells were treated with 2 µM, 5 µM, or 10 µM AS-1842856 for 48 h, unless otherwise noted. Dimethyl sulfoxide (DMSO) (Sigma #D2650) was used as vehicle for both compounds.

### Gene Silencing.

#### H3-3A.

*H3-3A* gene was knocked down in DIPG007 cells by using sh*H3-3A*-containing lentiviral particles generated according to the LentiStarter 3.0 kit (SBI #LV060-1, Palo Alto, CA). Briefly, HEK293T cells were transfected with 700 ng of two independent human sh*H3-3A* plasmids purchased from Sigma (clone IDs: TRCN0000139066 = sh1 or TRCN0000139180 = sh2). HEK293T-transfected media containing lentiviral particles were collected 72 h post transfection and filtered with a 0.45 µm-syringe filter. Next, the filtered lentiviral particles were added into media of previously plated DIPG007 cells, and 48 h post transduction, culture media was replaced by fresh growth media. After an additional 48 h, media was supplemented with 1 μg/mL puromycin for selection of successfully transduced cells.

#### FOXO1.

*FOXO1* gene was knocked down in DIPG007 cells by using sh*FOXO1*-containing lentiviral particles generated according to the TransIT® Lentivirus System (Mirus #MIR 6655). Briefly, HEK293T cells were transfected with human sh*FOXO1* plasmids purchased from Sigma (clone IDs: TRCN0000010333 or TRCN0000039580). HEK293T-transfected media containing lentiviral particles were collected 48 h post transfection and filtered with a 0.45 µm-syringe filter. Next, the filtered lentiviral particles were added into media of previously plated DIPG007 cells, and 48 h post transduction, culture media was replaced by fresh growth media. After an additional 48 h, media was supplemented with 1 μg/mL puromycin for selection of successfully transduced cells.

### Cell Lysate Extraction.

#### Histone extraction.

Briefly, cells were washed with phosphate-buffered saline (PBS) (Gibco #10010-023) and centrifuged. Cell pellets were resuspended with a hypotonic lysis buffer (10 mM Tris HCl pH8.0, 1 mM KCl, and 1.5 mM MgCl2) supplemented with protease (Sigma #P8340) and phosphatase (APExBIO #K1012) inhibitor cocktails and incubated on a rotator at 4 °C for 30 min for nucleus isolation. The isolated nuclei were pelleted under centrifugation, resuspended with sulfuric acid (0.4 N H2SO4), and incubated on a rotator at 4 °C overnight. Following centrifugation, the supernatant was transferred to a new tube, and trichloroacetic acid (Sigma #T0699) was added followed by a 30 min incubation on ice. Next, the histone mix was centrifuged, supernatant discarded, and purified histones in the tube were washed twice with ice-cold acetone under centrifugation (5 min/wash). After the last acetone wash, histones were air-dried at room temperature for 20 min and resuspended with double-distilled water.

#### Whole-cell protein lysate.

Cells were washed with PBS and centrifuged. Cell pellets were resuspended with RIPA lysis buffer (Thermo Fisher Scientific #8990) supplemented with protease (Sigma #P8340) and phosphatase (APExBIO #K1012) inhibitor cocktails and incubated on a rotator at 4 °C for 1 h. Next, the mix was centrifuged and the supernatant containing whole-cell protein was transferred to a new tube. Pierce™ BCA Protein Assay Kit (Thermo Fisher Scientific #23225) was used to quantitate both whole-cell protein lysate and extracted histones.

### Immunoblotting.

Whole-cell protein and histone lysates were resolved in 4 to 15% Mini-PROTEAN® TGX™ Precast Protein Gels (BIO-RAD #4561084) and transferred to a PVDF membrane by using the Trans-Blot Turbo RTA Mini 0.2 µm Transfer Kit (BIO-RAD #1704272). After membrane blocking with 5% milk in tris-buffered saline (TBS) supplemented with 0.1% Tween-20 (TBST) at room temperature for 1 h on a rocker, blots were incubated at 4 °C overnight with the following primary antibodies: (*Whole-cell protein lysate*) Brg1/SMARCA4 (Cell Signaling #52251), SMARCA2 (Bethyl #A301-015A), PBRM1 (Cell Signaling #91894), ARID2 (Novus Biologicals #NBP1-26615), BRD7 (Thermo Fisher Scientific #PA5-49379), SMARCB1 (Cell Signaling #91735), FOXO1 (Cell Signaling #2880), Phospho-FOXO1 (Ser256) (Cell Signaling #9461), HSP90 (Cell Signaling #4877), and β-actin (Sigma #A5441); (*histone*) H3K27Ac (Millipore #07-360), H3K4me1 (Cell Signaling #5326), H3K4me3 (Cell Signaling #9751), H3K27me3 (Cell Signaling #9733), H3.3K27M (Sigma #ABE419), and histone H3 (Cell Signaling #3638). Next, immunoblots were washed with TBST and incubated with either goat anti-rabbit IgG (H + L)-HRP conjugate (BIO-RAD #1706515) or goat anti-mouse IgG (H + L)-HRP conjugate (BIO-RAD #1706516) secondary antibodies at room temperature for 1 h. Then, after membranes were washed with TBST, immunoblot signals were detected by using either Pierce™ ECL Western Blotting Substrate (Thermo Fisher Scientific #32106) or SuperSignal™ West Pico PLUS Chemiluminescent Substrate (Thermo Fisher Scientific #34578) reagents on an autoradiography film (dotScientific #BDB57-Lite).

### Cell Viability.

Cell viability was assessed using the CellTiter-Glo® 2.0 Cell Viability Assay (Promega #G9242) according to manufacturer’s instructions. Briefly, 5,000 cells were seeded (in triplicate/condition) in a solid bottom white-walled 96-well plate and incubated with different concentrations of the drug for 5 d. Luminescence signal was detected using a BioTek Synergy HTX Multi-Mode microplate reader (Agilent company), and relative light unit (RLU) was analyzed with GraphPad Prism 8.4.3 (GraphPad software). Percentage of cell viability was calculated by dividing the RLU of drug-treated sample by the RLU of the control (drug vehicle) and multiplying by 100. Another method of cell viability analysis was by mixing cells with Trypan Blue Solution, 0.4% (Thermo Fisher Scientific #15250061), in a 1:1 dilution and inserting into a cell-counting chamber slide (Thermo Fisher Scientific #C10312) to read in a Countess™ 3 FL Automated Cell Counter (Thermo Fisher Scientific #AMQAF2000).

### Immunohistochemistry.

Immunohistochemistry was performed on H3K27M and H3WT DMG samples as previously reported (54, 55). The Discovery XT processor (Ventana Medical Systems) or Leica Bond automated staining processor (Leica Biosystems) was used to perform immunohistochemical staining. Each section from H3K27M or H3WT tumor sample was blocked using 10% normal goat serum along with 2% BSA in PBS for a duration of 30 min. After this, each section was treated with a rabbit monoclonal anti-SMARCA4 antibody (1:200, Abcam, ab110641) for a duration of 5 h. This was followed by treating individual tissue sections with biotinylated goat anti-rabbit IgG (PK6101, Vector Labs) at a dilution of 1:200 for a duration of 60 min. Chromogens were detected using the DAB detection kit along with Streptavidin-HRP and Blocker D to reduce background (Ventana Medical Systems). These treatments were performed according to instructions provided by the manufacturer. Subsequently, each section was mounted, dried, and visualized using the scanning system from Aperio Vista (AperioScanscope Scanner). Each slide was visualized through the accompanying AperioImageScope software program. We quantified slides in a blinded manner. An experimenter blinded to the study design visualized each slide at 40× magnification. They then captured images in JPEG format. JPEG images were captured from three randomly selected areas of each H3WT- and H3K27M-stained section. We used our previously published, automated analysis program to quantify each image. This program is MATLAB based and uses the image processing toolbox (54). K-means clustering, color segmentation based on RGB color differentiation, and Otsu’s threshold-based background–foreground separation are taken into consideration to arrive at a quantitative score that multiplies extracted pixels with the average intensity for each JPEG image.

### TMT Mass Spectrometry.

Whole-cell protein lysates were collected from DIPG007 cells from the following conditions: (a) untreated (n=3); (b) stably knocked down for H3-3A/H3F3A (H3-3AKD sh1) (n=3); (c) treated with AU-15330 PROTAC (1 μM for 24 h) (n=4); and (d) vehicle (Veh = DMSO for 24 h) (n=3). Samples were then sent to the Proteomics Resource Facility at the University of Michigan for analysis. Briefly, 100 μg of the lysate/sample/replicate was prepared and labeled following the directions of the TMTpro™ 16plex Label Reagent Set (Thermo Fisher Scientific #A44521). Twelve fractions were used for LC-MS/MS analysis. Generated data were aligned to a human protein database (20291 entries; reviewed; downloaded on 12/13/2021) and sorted for high (≤1%) or medium-low (≤5%) FDR confidence. A heatmap of protein abundance was generated using GraphPad Prism 8.4.3 (GraphPad software).

### RNA Sequencing.

DIPG007 cells were treated with 1 µM AU-15330 or DMSO for 24 h, and total RNA was extracted using Trizol (Thermo Fisher #15596026) followed by DNAse (Sigma #DN25) treatment. Preparation of complementary DNA (cDNA) library and sequencing were performed according to the Illumina TruSeq protocol and HiSeq 2000 platform, respectively. The resultant RNA-Seq data were aligned to human reference genome by using Bowtie software and analyzed with the RNA-Seq by Expectation-Maximization (RSEM) software tool. Sorting of differentially expressed genes was performed by using the empirical Bayes hierarchical models (EBSeq), and significantly up-regulated and down-regulated pathways were determined by the Molecular Signatures Database (MSigDB) in GSEA software (https://www.gsea-msigdb.org/gsea/msigdb/). RNA-Seq datasets have been deposited at the Gene Expression Omnibus (GEO) and an accession number is pending.

### ATAC Sequencing.

ATAC sequencing was performed by the epigenetic services of Active Motif company (Active Motif), and samples were prepared according to the company’s protocol. Briefly, following treatment with 1 µM AU-15330 or DMSO for 24 h, DIPG007 cells were centrifuged, resuspended, and incubated in growth media with DNase solution at 37 °C for 30 min. Next, cells were centrifuged, and the pellet was resuspended in ice-cold PBS. Following cell count, a volume accounting for 100,000 cells was transferred to two separate tubes (representing two biological replicates), which were centrifuged and supernatant discarded. The remaining cell pellet was resuspended in ice-cold cryopreservation solution (50% FBS, 40% growth media, and 10% DMSO) and stored in a −80 °C freezer until shipping. ATAC-Seq data were aligned to hg38 human reference genome. Comparative analysis was performed by a standard normalization method, and peaks were determined using the MACS 3.0.0 algorithm at a cutoff of *P*-value 1×10^7^, without control file, and with the -nomodel option. False peaks were removed according to the ENCODE blacklist.

### Statistical Analysis.

Data were analyzed and statistical tests were applied (unpaired Student’s *t* test or ANOVA) using GraphPad Prism 8.4.3 (GraphPad software). Venn diagrams were generated by Venny^2.1^ software (https://bioinfogp.cnb.csic.es/tools/venny/). Statistical significance is determined for *P*-value < 0.05; error bars represent ± SD.

## Supplementary Material

Appendix 01 (PDF)

Dataset S01 (XLSX)

Dataset S02 (XLSX)

Dataset S03 (XLSX)

Dataset S04 (XLSX)

Dataset S05 (XLSX)

## Data Availability

All study data are included in the article and/or *SI Appendix*. RNA-seq and ATAC-seq data have been deposited in the National Center for Biotechnology Information (NCBI)- Gene Expression Omnibus (GEO) under GSE229454 (56).
