# Supplementary material for: Targeting SWI/SNF ATPases in H3.3K27M diffuse intrinsic pontine gliomas
Source: Proc Natl Acad Sci U S A. 2023 Apr 24;120(18):e2221175120. doi: 10.1073/pnas.2221175120 (PMC10161095; doi:10.1073/pnas.2221175120)
Supplement: Supplementary file 1 — Appendix 01 (PDF) [file pnas.2221175120.sapp.pdf]

PNAS

# Targeting SWI/SNF ATPases in H3.3K27M Diffuse Intrinsic Pontine Gliomas

Mateus Mota[1-2], Stefan R. Sweha[1-2], Matt Pun[1-4], Siva Kumar Natarajan[1-2], Yujie Ding[1-2], Chan Chung[5], Debra Hawes[6], Fusheng Yang[6], Alexander R. Judkins[6], Susanta Samajdar[7], Xuhong Cao[8], Lanbo Xiao[8], Abhijit Parolia[8], Arul M. Chinnaiyan[8-11#], Sriram Venneti[1-3,8.9#]

1. Laboratory of Brain Tumor Metabolism and Epigenetics, Department of Pathology, University of Michigan, Ann Arbor, MI, USA.
2. Chad Carr Pediatric Tumor Center, Department of Pediatrics, University of Michigan, Ann Arbor, MI, USA.
3. Cellular and Molecular Biology Program, University of Michigan Medical School, Ann Arbor, MI, 48109, USA.
4. Medical Scientist Training Program, University of Michigan Medical School, Ann Arbor, MI, 48109, USA.
5. Department of New Biology, Daegu Gyeongbuk, Institute of Science and Technology (DGIST), Daegu 42988, Korea.
6. Department of Pathology and Laboratory Medicine, Children's Hospital Los Angeles, Keck School of Medicine University of Southern California, Los Angeles, CA, 90027, USA.
7. Aurigene Discovery Technologies, Bengaluru, Karnataka, India.
8. Michigan Center for Translational Pathology, Department of Pathology, University of Michigan Medical School, Ann Arbor, MI, 48109, USA.
9. Rogel Cancer Center, University of Michigan Medical School, Ann Arbor, MI, 48109, USA.
10. Department of Urology, University of Michigan Medical School, Ann Arbor, MI, 48109, USA.
11. Howard Hughes Medical Institute, University of Michigan Medical School, Ann Arbor, MI, 48109, USA.

**# Correspondence to:**
Arul M. Chinnaiyan, MD, PhD
Director, Michigan Center for Translational Pathology
S.P. Hicks Endowed Professor of Pathology
Investigator, Howard Hughes Medical Institute
University of Michigan
1500 E. Medical Center Drive
Ann Arbor, MI 48109
(734) 615-4062
arul@med.umich.edu

Sriram Venneti, MD, PhD
Associate Professor of Pathology and Pediatrics
University of Michigan
3520E MSRB 1, 1150 W. Medical Center

Ann Arbor, MI 41804
734-763-0674
svenneti@med.umich.edu

**This PDF includes:**

Figures S1 to S3

**Other supporting materials for this manuscript include the following:**

Datasets S1 to S5

Figure S1

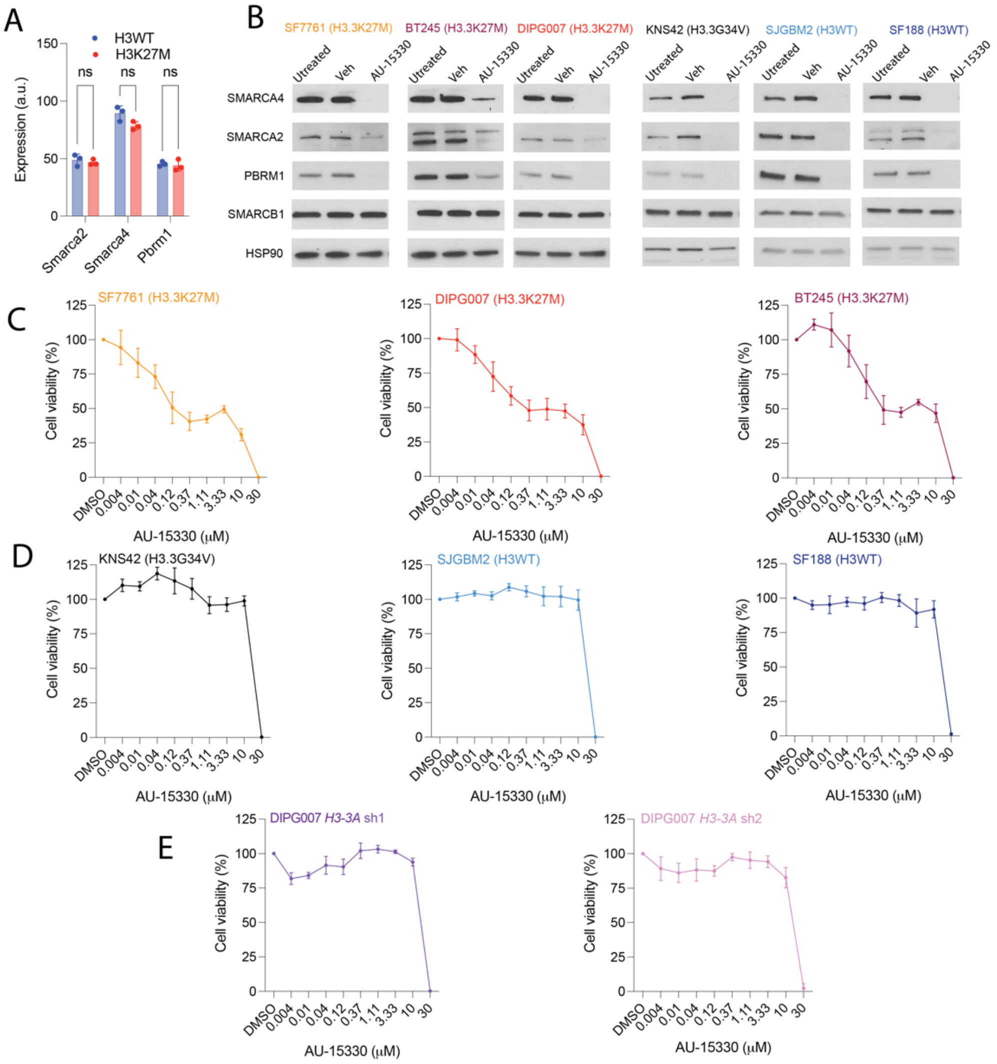


**Figure S1: H3.3K27M cell lines are sensitive to SMARCA2, SMARCA4, and PBRM1 protein degradation using the PROTAC AU-15330.**

**(A)** Gene expression of Smarca4, Smarca2, and Pbrm1 (Y-axis, a.u.=arbitrary units, n=3 each) in H3.3WT or H3.3K27M isogenic mouse neuronal stem cells (mNSC) from Chung et al. 2020; n.s. = non-significant**.**

**(B)** Immunoblots of H3.3K27M mutant cell lines (SF7761, BT245, and DIPG007), H3.3G34V mutant (KNS42), and H3WT (SJGBM2 and SF188) treated with AU-15330 (10μM for 24h) or Veh and probed for SMARCA4, SMARCA2, PBRM1, and SMARCB1. HSP90 was probed as loading control.

**(C)** Cell viability curves (normalized to Veh, percentage, Y-axis) of H3.3K27M mutant (SF7761, BT245, and DIPG007) cells upon treatment with different concentrations of AU-15330 (μM, X-axis) for five days (n=3 for each concentration/cell line).

**(D)** Cell viability curves (normalized to Veh, percentage, Y-axis) of H3WT (SJGBM2 and SF188) and H3.3G34V mutant (KNS42) cells upon treatment with different concentrations of AU-15330 (μM, X-axis) for five days (n=3 for each concentration/cell line).

**(E)** Cell viability curves (normalized to Veh, percentage, Y-axis) of DIPG007 cells with *H3F3A* KD sh1 and sh2 cell lines upon treatment with different concentrations of AU-15330 (mM, X-axis) for five days (n=3 for each concentration/cell line).

Figure S2

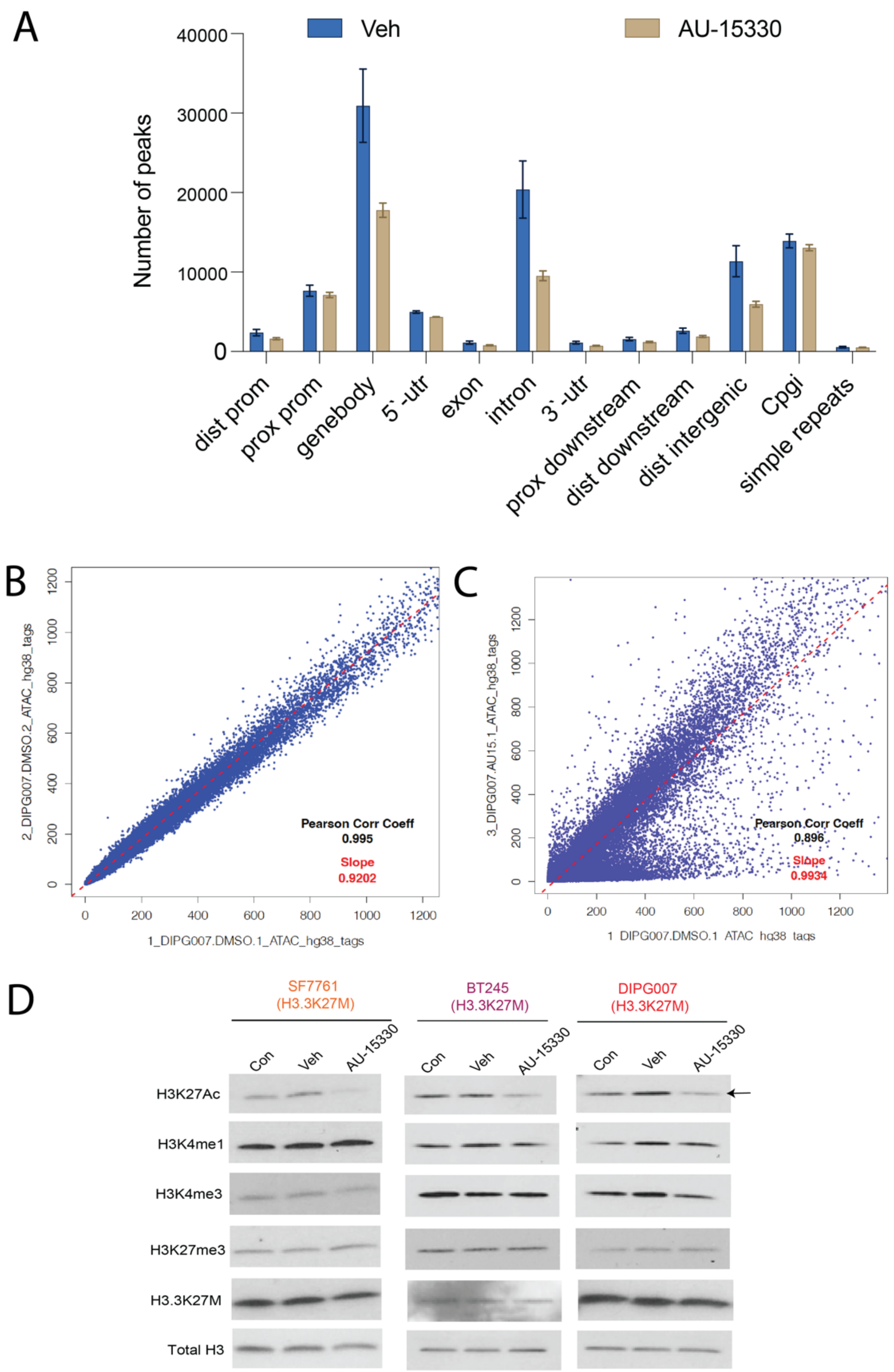


**Figure S2: AU-15330 reduces chromatin accessibility at non-promoter regions of H3.3K27M mutant cells.**

**(A)** Number of annotated ATAC-seq peaks in DIPG007 cells treated with AU-15330 (1µM for 24h) or Veh (n=2, each).
**(B)** Scatter plot of ATAC-seq peak values comparing DIPG cells treated with Veh (replicate 1 vs. replicate 2).
**(C)** Scatter plot of ATAC-seq peak values comparing DIPG cells treated with AU-15330 (1µM for 24h) vs Veh.
**(D)** Immunoblots of H3.3K27M mutant cell lines (SF7761, BT245, and DIPG007) untreated or treated with Veh or AU-15330 (10µM for 24h) and probed for H3K27Ac, H3K4me1, H3K4me3, H3.3K27M, and H3K27me3. Total H3 was probed as loading control. Arrow indicates H3K27ac.

Figure S3

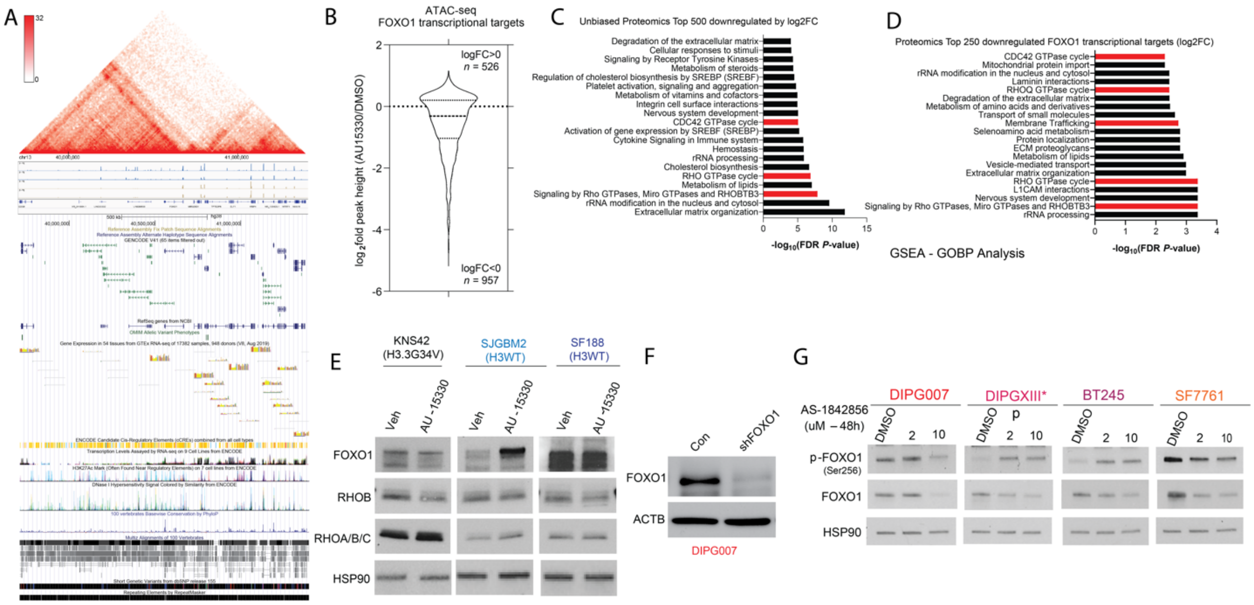


**Figure S3: Chromatin accessibility, gene expression, and protein abundance of FOXO1 are lowered by AU-15330 PROTAC.**
**(A)** Hi-C heatmap depicting a topologically associating domain (TAD) formed between the *FOXO1* promoter and ATAC-seq peaks 1 and 2 associated with the *FOXO1* locus in DIPG007 cells. Complete alignment of the TAD is depicted.
**(B)** Violin plot showing $log_2$fold change of ATAC-seq peak heights at FOXO1 transcriptional targets between AU-15330 (1µM for 24h) and vehicle-treated DIPG-007 cells.
**(C)** Pathway analysis of top 500 downregulated proteins by unbiased proteomics in AU-15330 vs. vehicle treated DIPG007 cells.
**(D)** Pathway analysis of top 250 downregulated FOXO1 transcriptional targets in proteomics dataset in AU-15330 vs. vehicle-treated DIPG007 cells.
**(E)** Immunoblots of H3.3G34V mutant (KNS42) and H3WT (SJGBM2 and SF188) cell lines treated with AU-15330 (1µM for 24h) or Veh and probed for FOXO1, RHOB, and RHOA/B/C. HSP90 was probed as loading control.
**(F)** Immunoblots in DIPG007 cells with or without *FOXO1* KD by shRNA probed for FOXO1. β-actin (ACTB) was probed as loading control.

**(G)** H3.3K27M mutant cell lines (SF7761, BT245, and DIPG007) were treated with vehicle (DMSO) or indicated doses of FOXO1 inhibitor AS-1842856 (that directly bind to the active FOXO1, but not the Ser256-phosphorylated form) for 48h. Cells were probed for FOXO1, p-ser256-FOXO1. HSP90 was probed as loading control.

**Datasets**

1. **Dataset S1.** ATAC-seq data from DIPG007 cells treated with AU-15330 (1μM for 24h) or Veh (n=2, each).
2. **Dataset S2.** RNA-seq data from DIPG007 cells treated with AU-15330 (1μM for 24h) or Veh (n=2, each).
3. **Dataset S3.** Proteomics data from DIPG007 cells treated with AU-15330 (1μM for 24h, n=4) or Veh (n=3, each).
4. **Dataset S4.** Gene lists corresponding to Venn diagram in Fig 3E.
5. **Dataset S5.** Gene lists corresponding to Venn diagram in Fig 4A.
